# Habit Reversal Therapy in Obsessive Compulsive Related Disorders: A Systematic Review of the Evidence and CONSORT Evaluation of Randomized Controlled Trials

**DOI:** 10.3389/fnbeh.2019.00079

**Published:** 2019-04-24

**Authors:** Melissa T. Lee, Davis N. Mpavaenda, Naomi A. Fineberg

**Affiliations:** ^1^Hertfordshire Partnership University NHS Foundation Trust, Welwyn Garden City, United Kingdom; ^2^University of Hertfordshire, Hatfield, United Kingdom

**Keywords:** habit reversal therapy, randomized controlled trial, obsessive compulsive and related disorders, HRT, RCT, habit

## Abstract

**Background:** Habit Reversal Therapy (HRT) has long been used in the treatment of Tourette Syndrome and Tic Disorders. It has more recently been used to treat Trichotillomania and skin picking behaviors, both considered as Obsessive Compulsive Related Disorders (OCRD).

**Objectives:** This literature review sought to establish and quality assess the existing randomized controlled trial evidence supporting the use of HRT in the DSM-5 family of OCRDs.

**Search Methods:** EMBASE, PsycINFO, PubMed, and Cochrane databases were searched for key terms relating to each OCRD (as classified in the DSM-5), and HRT.

**Selection Criteria:** Titles and abstracts were screened, and any literature matching pre-specified criteria were then selected to be reviewed further. Of these, 8 Randomized Controlled Trials (RCT) relating to Trichotillomania, and 2 RCTs relating to Excoriation Disorder, were extracted and reviewed against the 2010 Consolidating Standards of Reporting Trials (CONSORT) statement.

**Results:** The review identified 10 RCTs of HRT, but these were limited to patients with a primary diagnosis of Trichotillomania or “excoriation behavior,” only. There were some reports of the use of HRT in Tourette Syndrome or Tic Disorder with secondary OCD, but the OCD symptoms were not reliably reported on.

**Conclusion:** There is a gap in the current literature regarding the use of HRT in the DSM-5 OCRDs. In those RCTs that have been reported, the quality of study methodology was questionable as evaluated by CONSORT criteria. The implications of these findings are discussed, and suggestions are made for future research.

## Introduction

Obsessive-compulsive disorder (OCD) is a common, relatively treatment refractory neuropsychiatric disorder. One of its cardinal symptoms involves the urge-driven performance of compulsions i.e., stereotyped, repetitive motor and mental acts, usually designed to avert harmful consequences. Established treatments include medication with selective serotonin reuptake inhibitors (SSRI), and Cognitive Behavior Therapy (CBT) with exposure and response prevention (ERP). Although many cases are improved with these treatments, rates of incomplete recovery and treatment resistance to standard therapies are high: Approximately 40% patients fail to respond and 50% need further treatment (Fineberg et al., 2015).

ERP can be difficult for patients to undertake successfully, as the treatment requires facing feared situations for prolonged periods without engaging in compulsions whilst waiting for the compulsive urges to abate. Consequently, adherence is reported to be relatively low and premature discontinuation rates high (McDonald et al., 1988; Abramowitz et al., 2002). Delayed treatment is known to prolong ill health and reduce therapeutic gain (Dell'Osso et al., 2013). New, more highly efficacious—and acceptable—treatment paradigms are therefore required to advance the clinical therapeutics of OCD.

Disorders other than OCD have been classified under the Obsessive Compulsive Related Disorders (OCRDs) grouping in the American Psychiatric Association (2013), Diagnostic and Statistical Manual of Mental Disorders (5th ed.) (DSM-5). These include Body Dysmorphic Disorder (BDD), Hoarding Disorder, Trichotillomania (hair-pulling disorder) and Excoriation Disorder. Compared with OCD, these OCRDs have received relatively little interventional analysis. Standard treatment approaches for BDD are similar to OCD and include SSRIs and CBT (Hong et al., 2018). Hoarding disorder is notably resistant to most forms of treatment (e.g., Ayers et al., 2014). In Trichotillomania and Excoriation Disorder, repetitive, stereotyped grooming acts represent key symptoms, and these OCRDs could otherwise be viewed as body-focussed habit disorders (Stein et al., 2006).

Meanwhile, advances are being made in understanding the neurobiological mechanisms underpinning OCD and the other OCRDs that may lead to the discovery and development of new therapeutic interventions (Fineberg et al., 2018). Many patients with OCRD describe their compulsions as being habitual in nature. Habits, otherwise known as “stimulus response behaviors,” are relatively fixed responses that, through habit learning, automatically occur in response to a particular environmental trigger. In behavioral terms, they are defined as being insensitive to changes either in the “outcome value” of the behavior or the “environmental contingency.” They are defined as “learned sequences of acts that have become automatic responses to specific cues, and are not functional in obtaining goals or end states” (Verplanken and Aarts, 1999, p. 104). In other words, once learned, habits (rather like compulsions) continue relatively unchanged, whether they are adaptive or not (Balleine and O'doherty, 2010). Over-reliance on the habit system has been hypothesized to play a role in generating compulsive symptoms in a range of OCRDs, such as OCD, trichotillomania (hair pulling disorder), excoriation (skin picking disorder), transient or motor tic disorder, and Tourette Syndrome (Chamberlain et al., 2007, 2009) and disorders of addiction (Voon et al., 2015; Gillan et al., 2016).

According to the “dual-system” theory, instrumental actions are normally supported both by a goal-directed system and a habit system, in dynamic balance (Balleine and O'doherty, 2010). According to this theory, the goal-directed system drives actions that are performed either to achieve desirable goals, or to avoid undesirable outcomes (Gillan et al., 2011). However, the habit system may take over once an action has been performed multiple times, exerting dominant control, so that the behavior is produced automatically without too much conscious effort. Although habit induction can be seen to lead to greater cognitive efficiency, it also leads to a loss of behavioral flexibility, and may thus contribute to the ongoing performance of stereotyped compulsive acts.

A rational next step, therefore, is to investigate whether treatments known to challenge habitual behavior are also effective in OCD and related disorders. The Habit Reversal Procedure was originally developed as a treatment for nervous habits and tics (Azrin and Nunn, 1973). Habit Reversal included several behavioral components that aim to help patients challenge habit performance, such as recording, awareness training, competing-response practice, habit- control motivation, and generalization-training. Habit Reversal Therapy (HRT) has since been refined and developed as a multi-component behavioral intervention (Woods, 2001), and is mainly used for the treatment of Tourette Syndrome (Woods and Miltenberger, 1995) and Tic Disorders. Of note, approximately 50% of those with Tourette syndrome experience obsessive-compulsive behaviors or diagnosable OCD at some point in their lifetime (Leckman et al., 1995). Moreover, in the treatment of Tourette Syndrome, it has been suggested that it is often the symptoms of related or comorbid conditions, such as obsessive-compulsive symptoms, and not the tics themselves, that require most attention (Goodman et al., 2006). Given its success in treating Tourette Syndrome and Tic Disorder and the acknowledged clinical and neurobiological overlap between OCD and Tourette Syndrome and Tic Disorders (Fineberg et al., 2010), it is logical to speculate that HRT would be effective in some cases of OCRD. Indeed in some forms of OCD, particularly in those patients with symmetry/ordering symptoms, patients report premonitory urges very like those seen in tic disorders (Subira et al., 2015). These findings suggest a potential role for HRT in OCD with comorbid tics or where symmetry/ordering symptoms are present.

Whereas, the existing neuropsychological evidence implicates habit as a key mechanism in OCD (Gillan et al., 2015), HRT has so far mainly been investigated in other disorders characterized by habits. To date, there is very little published data on the effect of HRT on obsessive compulsive symptoms in OCD (reviewed in Coffey and Rapoport, 2010). HRT is one of the few treatments thought to be effective in Trichotillomania, together with SSRIs and other forms of behavioral therapy (Chamberlain et al., 2007). In Trichotillomania, compulsive hair pulling, affecting various parts of the body and resulting in noticeable hair loss, is associated with considerable shame and distress (Drysdale et al., 2009). The peak age at onset is 12–13 years, with the disorder often being chronic and difficult to treat (Diefenbach et al., 2000; Walsh and McDougle, 2001). Medical complications can arise in addition to the cosmetic and psychosocial consequences of the disorder, including infection, repetitive stress injury, and permanent loss of hair (Frey et al., 2005). Therefore, treatment studies should ideally investigate young people with trichotillomania as well as adults, and explore the effects of treatment on mental and physical health comorbidities.

A recent meta-analysis analysis (Bate et al., 2011) demonstrated a large effect of HRT from pre-assessment to final post-treatment assessments, in a combined group of disorders including Trichotillomania, Tourette Syndrome, nail biting, and stuttering, amongst other habitual behaviors. Research is continuing to support the use of HRT in Trichotillomania (e.g., Rahman et al., 2017), and has started to expand to look at its effectiveness in Excoriation Disorder, alongside a few other treatments thought be of benefit, such as SSRIs, glutamatergic agents and CBT (Lochner et al., 2017). Such research has used HRT in various modified forms. For example, Acceptance Enhanced Behavior Therapy (AEBT), in which Acceptance and Commitment Therapy (ACT) is combined with HRT, was used with some success in a case series of patients with Excoriation Disorder (Capriotti et al., 2015).

Given the increasing evidence suggesting the effectiveness of HRT for Trichotillomania, a disorder classified as an OCRD in the DSM-5, the question arises as to whether HRT would also work for other OCRDs, namely OCD, BDD, Hoarding Disorder and Excoriation Disorder. This literature review seeks to start to answer this question by establishing the research that has been conducted into the use of HRT for patients across the whole range of OCRDs and expands on this further by assessing the quality of any randomized controlled trials (RCTs) identified from this search using the standard Consolidated Standards of Reporting Trials (CONSORT) criteria (Moher et al., 2010).

## Methodology

### Identification and Selection of Studies

A computer search was conducted using the following three databases: EMBASE, PsycInfo, and PubMed. Narrow searches were conducted using variable terms for “Habit Reversal Therapy,” and the following OCRDs: OCD, BDD, Hoarding Disorder, Trichotillomania, and Excoriation. The Boolean operators “AND” and “OR” were used to combine these terms, and search in the title and abstract of available papers. There was no lower limit to the time period for publication and searches continued until March 2018. Please refer to Appendix 1 in Supplementary Material for a full table of search terms used.

The titles and abstracts of papers were then screened for suitability, by looking for key words and phrases that included “habit reversal training,” “habit reversal therapy,” and the OCRDs. Exclusion criteria were those that did not have “habit reversal” in addition to an OCRD in their title and/or abstract, and those that were not available in English language. Any experimental studies were screened for the use of HRT within their methodology in some way, for example a combined therapeutic approach, or stating its use within a “behavior therapy,” for example. Due to the number of “hits” for Tourette Syndrome and Tic Disorders with comorbid OCD, the decision was made to only include papers looking at these primary disorders that stated “Obsessive Compulsive Disorder” and exclude those that referred to “Obsessive Compulsive Symptoms.” Where abstracts were not available, the full text was screened according to the same criteria.

**Figure 1:**
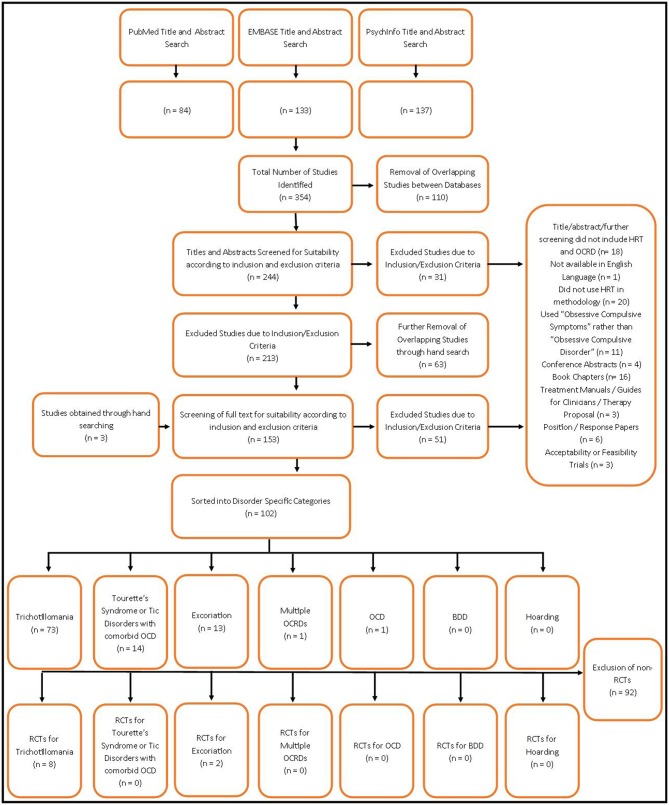
Literature review flowchart.

A hand search was then conducted to screen references for any additional studies that may have been missed in this process. A separate search of the Cochrane Library was also performed. Conference abstracts, book chapters, treatment manuals, position papers, surveys, and acceptability trials were excluded from any results at any stage of the search. For a summary of this process, please refer to Figure 1.

Papers identified by the process outlined above were then screened for inclusion in the CONSORT evaluation. Only those studies identified as a randomized controlled study (RCT) from reading the title, abstract, or methodology, were included in this quality analysis.

### Quality Assessment

The quality of the included studies was assessed by the 25-item version of the CONSORT (Consolidating Standards of Reporting Trials) statement (Schulz et al., 2010), using the guidance published by Moher et al. (2010). The CONSORT statement is primarily utilized to assess RCTs, but it has been extended to cover other designs, such as non-inferiority and equivalence trials, and reporting of harm-related data (Boutron et al., 2008). The checklist, published in 1996 and revised in 2001, 2008, and 2010, comprises a set of guidelines that may be used to identify the strengths and weaknesses of clinical trials for both pharmacologic and non-pharmacologic treatments (Des Jarlais et al., 2004; Schulz et al., 2010). For example, in regard to study methodology, the checklist assesses whether a study has adequately reported the eligibility criteria for participants, has provided the precise details of the interventions intended for each group, and has provided justification (e.g., power analysis) for the obtained sample size. Failure to report these details will result in a lower level of CONSORT compliance and thus lower overall reporting quality.

All included studies were assessed for compliance with the 2010 guidelines of the CONSORT statement. To measure compliance, as advised within the CONSORT statement, a two-point grading system was applied for each CONSORT criterion, where the first author (ML) gave a score of “0” if the item was not present at all, a “1” if the feature was partially present (i.e., some aspects of the CONSORT item were missing or unclear), and a “2” if the CONSORT item was present and clear. To demonstrate this scoring method, the CONSORT item 3 states: “Eligibility criteria for participants and the settings and locations where the data were collected.” A score of 0 on this item would be given if the researchers noted that eligibility criteria were used but did not explain what these criteria were, and did not report the settings and locations where the data were collected; a score of “1” would be given if the researchers provided complete details of the eligibility criteria (inclusion and exclusion criteria), but did not report the settings and locations where the data were collected (or vice versa); and a score of “2” would be given if the researchers provided clear descriptions of both the eligibility criteria used in the study and the setting and locations where the data were collected. In instances where the CONSORT item was not present due to inherent limitations of the study design (e.g., stating who was blinded after assignment to interventions), a score of “0” on that item was given. By systematically applying the CONSORT criteria to all relevant sections of each study, an overall summary of the study's quality as a clinical trial was produced. The evaluation method was independently checked for validity and consistency by one of the two co-authors, who repeated the exercise without knowledge of the original evaluation third author (DM). Where there was disagreement, both raters blindly re-rated the specific items, discussed the ratings, and reached an agreement.

## Results

For clarity, we present the results of the literature review according to each OCRD, followed by the results of the CONSORT evaluation of all the identified Randomized Controlled Trials (RCTs).

### Literature Review

#### Habit Reversal Therapy in Trichotillomania

Overall, the literature search identified 30 case studies or series, 21 literature reviews, 1 systematic review/meta-analysis, 1 meta-analysis, 8 RCTs, and 12 other trials of HRT in hair pulling behavior and/or Trichotillomania. The 8 RCTS were each small in number of participants (after dropouts were considered, maximum *N* = 40, Rahman et al., 2017) and limited in several other aspects of design. Only 2 RCTs involved young people (Azrin et al., 1980; Rahman et al., 2017). They each found some evidence of benefit for HRT, mainly limited to the severity of hair pulling. Importantly, few studies reported using “Habit Reversal” as the defined experimental intervention; many termed the intervention as CBT but stated in the methodology that this contained components of HRT (see individual studies and meta-analyses below). Specifically, two RCTs stated that they used HRT on its own at some stage within the intervention (Azrin et al., 1980; Rahman et al., 2017). Two RCTs used HRT combined with another approach (Woods et al., 2006; Shareh, 2018), and another two used HRT as a component of an experimental intervention (Moritz and Rufer, 2011; Keuthen et al., 2012). The final two RCTs were designed to compare the effect of augmenting HRT with a pharmacological intervention (Ninan et al., 2000; Dougherty et al., 2006). The choice of control intervention (e.g., wait list; no treatment) was inadequate in many of the studies; notwithstanding, relatively high “placebo-response” rates were found in some studies employing a form of neutral control (e.g., Azrin et al., 1980), emphasizing the importance of using an adequate comparison group to judge effect size. In addition, there was lack of consistency in the use of assessment instruments and some of the studies relied on a completer analysis, resulting in an increased risk of bias toward treatment success.

Please refer to Table 1 for a list of the randomized controlled trials directly investigating the efficacy of HRT.

**Table 1:** Randomized controlled trials of HRT in trichotillomania.

**Study/country**	**Assessment of disorder**	**Excluded comorbidity**	**Interventions**	**No. HRT/BT sessions**	***N***	**Age mean/(range) in years**	**Outcome measures**	**Duration of the trial (baseline to end point)**	**Follow-up, after end point?**	**ITT analysis? Yes/No**	**Outcome at endpoint**	**Treatment responders (%)**
(Azrin et al., 1980)/USA	Self-report and visual inspection of hair loss	N/R	1. HRT 2. NPP	1 session of HRT + telephone contact over 2–3 days	34	28	Self-report frequency and duration of hair pulling/; family/friend report of hairpuling frequency.	Up to 3 days	Yes; daily for first week, weekly for first month, monthly to 4 months. 22 months for HRT only.	No	HRT significantly better than NPP and each of the follow up periods (p = < 0.05).	N/R
(Ninan et al., 2000)/USA	DSM-III-R	N/R	1. CBT 2. Clomipramine 3. Placebo	9	23	33.38 (22–53)	NIMH-TSS; NIMH-TIS; CGI-I; BDI; STAI	9 weeks	No	Not clear	CBT significantly better than clomipramine and placebo on TSS and TIS (both *p* < 0.05).	100% of completers, 71% of intent-to-treat for HRT group
(Dougherty et al., 2006)/USA	DSM-IV	Suicidal or homicidal risk, bipolar disorder, psychosis, organic mental disorder, developmental disorder.	1.12 weeks Sertraline, followed by HRT for non-responders 2. 12 weeks Placebo, followed by HRT for non-responders	2	24	1.31.5 2. 26.3	HPS; PITS; TTMIS; CGI; HAM-D; BDI; BAI; Q-LES-Q	22 weeks	N/R	Yes	Significant group differences on HPS (*p* = 0.017) and CGI (*p* = 0.026) comparing sertraline + HRT vs. sertraline or HRT monotherapies analyzed as a single group.	(Sertraline and HRT) = 54.5%; (HRT or Sertraline) = 15.4%.
(Woods et al., 2006)/USA	DSM-IV	Schizophrenia, MDD, or another disorder requiring immediate attention	1. ACT/HRT 2. WL	10 session; 8 weekly, 2 bi-weekly	25	35	MGH-HPS; NIMH-TIS; Self-monitoring of pulling; PAI; AAQ; TEI-SF; NIMH clinician impairment rating	12 weeks	Yes; 3 months	No	ACT/HRT significantly better than WL on MGH-HPS (*p* < 0.01), NIMH clinician impairment rating (*p* < 0.05), and self-reports of hair pulling (*p* < 0.01).	N/R
(Moritz and Rufer, 2011)/Online	Self-report of diagnosis received	Psychosis, Bipolar Disorder	1. Self-help DC (partially a variant of HRT) 2. Self-help PMR	N/R	42	1.31.5 2. 29.4	MGH-HPS; OCI-R; BDI-SF	4 weeks	No	Yes	DC significantly better than PMR on MGH-HPS (*p* = 0.05); significant within-group improvement in OCI-R for DC (*p* = 004), but not PMR.	N/R
(Keuthen et al., 2012)/USA	DSM-IV	Serious psychiatric disorders, including psychosis, ADHD, lifetime alcohol or substance dependance.	1. DBT/CBT 2. MAC	11 weekly 50-min sessions	38	30.71	NIMH-TSS; NIMH-TIS; CGI; MGH-HPS; DERS; NMR; ARR; BDI-II; BAI; AAQ; CSF	12 weeks	Yes; 3 and 6 months	No	DBT/CBT significantly better than MAC on between group analysis using MGH-HPS (*p* < 0.001); NIMH-TSS (*p* < 0.001); NIMH-TIS (*p* < 0.001); ARR (*p* < 0.001).	11 DBT/CBT participants and 1 MAC participant
(Rahman et al., 2017)/USA	DSM-IV	Bipolar disorder, psychotic disorder, autism spectrum disorder.	1. HRT 2. TAU	8 weekly 50-min sessions	40	(7–17)	ADIS-IV-C/P; MGH-HPS; NIMH-TSS; CGI; TDI; SACA; CDI; MASC	9–10 weeks	Yes; 1 and 3-month treatment responders only	No	HRT significantly better than TAU on between group analysis using NIMH-TSS (*p* < 0.001) and MGH-PS (*p* < 0.002). Significantly greater number of responders in HRT vs. TAU group (*p* < 0.001).	76% in HRT, 21% in TAU
(Shareh, 2018)^*^/Iran	DSM-5	Psychotic, neurological disease or substance abuse, psychological and personality disorders (not including GAD, dysthymia, and MDD).	1. MCT/HRT 2. WL	8 weekly sessions	38	MCT/HRT = 32.06 WL = 31.14^*^	SCID-I/P; SCID-II; PITS; MGH-HPS; Y-BOCS-TM; WASI; BDI-II; BAI; RSES; self-monitoring; GAF; CGI; CSQ; WAI-S.	8 weeks	No	No	MCT/HRT significantly better than WL on BDI-II (*p* < 0.001); BAI (*p* < 0.001); RSES (*p* < 0.001); self-monitoring (*p* < 0.001); MGH-HPS (*p* < 0.001); Y-BOCS-TM (*p* < 0.001); GAF (*p* < 0.001).	N/R

AAQ, Acceptance and Action Questionnaire; ACT/HRT, Acceptance and Commitment Therapy with Habit Reversal Therapy; ADIS-IV-C/P, Anxiety Disorders Interview Scheduled for DSM-IV: Child and Parent Versions; ARR, Affective Regulation Rating; BAI, Beck Anxiety Inventory; BDI, Beck's Depression Inventory; BDI-II, Beck Depression Inventory-Second Edition; BDI-SF, Beck Depression Inventory Short Form; BT, Behavior Therapy; DBT, Cognitive Behavioral Therapy; CDI, Children's Depression Inventory; CGI, Clinical Global Improvement Scale; CGI-I, Clinical Global Impressions-Improvement; CSF, Consumer Satisfaction Form; CSQ, Client Satisfaction Questionnaire; DBT/CBT, Dialectical Behavior Therapy Enhanced with Cognitive Behavioral Therapy; DC, Decoupling; DERS, Difficulty in Emotion Regulation Scale; DSM-III-R, Diagnostic and Statistical Manual of Mental Disorders, Third Edition, Revised; DSM-IV, Diagnostic and Statistical Manual of Mental Disorders, 4th Edition; DSM-5, Diagnostic and Statistical Manual of Mental Disorders, Fifth Edition; GAD, Generalized Anxiety Disorder; GAF, Global Assessment of Functioning; HAM-D, 17-Item Hamilton Rating Scale for Depression; HPS, Hair Pulling Scale; HRT, Habit Reversal Therapy; ITT, Intent to Treat Analysis; MAC, Minimal Attention Control; MASC, Multidimensional Anxiety Scale for Children; MCT/HRT, Metacognitive Methods combined with Habit Reversal Therapy; MDD, Major Depressive Disorder; MGH-HPS, Massachusetts General Hospital Hairpulling Scale; NIMH-TIS, National Institute of Mental Health Trichotillomania Impairment Scale; NIMH-TSS, National Institute of Mental Health Trichotillomania Severity Scale; NMR, Negative Mood Regulation Scale; NPP, Negative Practice Program; N/R, Not recorded; OCI-R, Obsessive-Compulsive Inventory-Revised; PAI, Personality Assessment Inventory; PITS, Psychiatric Institute Trichotillomania Scale; PMR, Progressive Muscle Relaxation; Q-LES-Q, Quality of Life Enjoyment and Satisfaction Questionnaire; RSES, Rosenberg Self-Esteem Scale; SACA, The Service Assessment for Children and Adolescents; SCID-II, Structured Clinical Interview for DSM-IV Axis II Disorders; SCID-I/P, Structured Clinical Interview for DSM-IV Axis I Disorders-Patient Edition; STAI, State-Trait Anxiety Inventory; TAU, Treatment as Usual; TDI, Trichotillomania Diagnostic Interview; Scale; TEI-SF, Modified Treatment Evaluation Inventory-Short Form; TTMIS, Trichotillomania Impact Scale; WAI-S, Working Alliance Inventory-Short; WASI, Wechsler Abbreviated Scale of Intelligence; WL, Wait List; Y-BOCS-TM, Yale-Brown Obsessive Compulsive Scale-Trichotillomania version;

* *indicates that RCT section of study only is reported in this table*.

#### Individual Randomized Controlled Trials

The first published RCT of HRT for “hair pulling behavior” was conducted by Azrin et al. (1980). In this pioneering study, a mixture of 30 adults and 4 children were recruited through a newspaper advertisement for treatment of hair pulling, with no formal diagnosis of Trichotillomania required for participation. A coin flip was used to assign participants to the Habit Reversal (19 participants) or a Negative Practice Treatment (15 participants) group. The Negative Practice Treatment involved the participants standing in front of a mirror and acting out the motions of hairpulling without doing any damage at various time intervals. HRT was delivered in one session of approximately 2 h duration, with telephone contact over the next 2 or 3 days, with calls decreasing in frequency as the hair pulling episodes decreased. No formal measures of hair pulling behavior or Trichotillomania symptoms were used; participants used a self-report chart of the number of occurrences or duration of hair pulling episodes, in addition to family or friend's self-report of observed frequency of hair pulling. Significant differences between the two groups were found in hair pulling behavior immediately after HRT and at each of the follow up periods, up to 3 months. For those in the HRT groups, hair pulling behavior reduced by 99% the first day after training, and 99% on the second day. The level of reduction (97–99.9%) was reported to continue for 4 weeks, and during the 4th month still reached 91%. This was compared to 58% reduction on the first day for the negative practice group, which produced 52–68% improvement during the 3-month follow-up.

In the study by Ninan et al. (2000), 23 adult participants with DSM-III-R Trichotillomania were randomized to receive CBT with HRT, Clomipramine, or pill placebo. Analysis indicated that those allocated to CBT underwent significantly greater change in severity (as measured by the Trichotillomania Severity Scale) and impairment (as measured by the Trichotillomania Impairment Scale), compared to both the Clomipramine and pill placebo groups at the 9-week end point. However, there was a high drop-out rate over the course of the study (4 of those receiving clomipramine, 2 receiving CBT, and 1 receiving placebo), reducing the sample size to 16 completers, on whom it appeared that the analysis was conducted. In addition, by omitting a psychological placebo treatment, the study was unable to adequately control for the nonspecific effects of therapist contact in the HRT group.

Dougherty et al. (2006) took a different approach, randomizing adult patients with DSM-IV Trichotillomania who had failed to respond to either 12 weeks of sertraline, or placebo, to 2 sessions of additional HRT. Those receiving HRT in combination with sertraline (*N* = 11) obtained better outcomes than those receiving HRT with placebo (*N* = 9), hinting at an added advantage for combining SSRI with HRT in trichotillomania. However, the design of the study means that it is difficult to draw conclusions on the effectiveness of HRT.

Woods et al. (2006) randomized 25 Caucasian women with Trichotillomania to a combined therapy involving 10 sessions of Acceptance and Commitment Therapy and Habit Reversal Therapy (ACT/HRT) or a waiting list condition. Standardized outcome measures were used, and a significant group by time interaction was found, with the ACT/HRT group showing a significant decrease in symptom scores across time and superior efficacy on self-report measures of hair pulling severity and impairment on the NIMH clinician impairment. These reductions were maintained at 3 months follow up.

Notably, in the study by Moritz and Rufer (2011), obsessive-compulsive symptomatology was measured (using the Obsessive Compulsive Inventory-Revised (OCI-R; Foa et al., 2002) alongside hair pulling in approximately 31 adults with a self-report diagnosis of Trichotillomania. The study compared “self-help decoupling” (authors stating that parts of decoupling may be considered a variant of HRT, as they both interfere at the motor level of functioning) with “self-help progressive muscle relaxation (PMR).” Using an intention to treat analysis, borderline significant advantage was found in favor of HRT in regards to hair pulling symptoms on the Massachusetts General Hospital-Hair-Pulling Scale (MGH-HPS) (*p* = 0.05), with medium to strong effect size. Decoupling was also associated with significant within-group improvements on measures of obsessive-compulsive symptoms (OCI-R; *p* = 0.04), hinting that HRT may have a therapeutic role in other OCRDs as well as trichotillomania.

In a sample of 33 adult participants with Trichotillomania, which excluded those with a history of treatment non-response, Keuthen et al. (2012) compared Dialectical Behavior Therapy Enhanced Cognitive Behavioral Treatment (DBT/CBT) to Minimal Attention Control (MAC). The DBT/CBT was stated to encompass “standard habit reversal training.” The DBT/CBT group received 11 acute treatment sessions, followed by 4 maintenance treatment sessions over the following 3 months, with follow up assessment conducted at 3 and 6 months after the acute sessions ended. There were significant between group differences in hair pulling severity (MGH-HPS); NIMH Trichotillomania Severity Scale (NIMH-TSS); and impairment (NIMH Trichotillomania Impairment Scale (NIMH-TIS), as well as in measures of Affective Regulation Rating (ARR) for completers on baseline to week 11 change scores. During follow-up, although MGH-HPS scores worsened from post-treatment to 3- and 6-month follow-up, significant improvement compared to baseline on all TTM variables was still reported at 3- and 6-month follow up in the DBT/CBT completer group.

Rahman et al. (2017) randomized 40 children and adolescents with a primary diagnosis of Trichotillomania to a treatment as usual (TAU) control group or a course of 8 weekly sessions of HRT (based on the treatment protocol outlined by Woods, 2001). A large between group difference (*d* = 0.87) at the study endpoint was reported using measures of hair pulling symptoms (NIMH-TSS; MGH-HPS). A significantly greater number of responders was found in the HRT compared to the TAU group. Notably, however 52% of those randomized to the TAU group received no treatment over the course of the study, and the TAU treatments were not standardized, thereby potentially biasing the outcomes in favor of the HRT group. Many (at least 75%) of those classed as a “treatment responder” on HRT who completed a 1- and 3-month follow up assessment maintained a treatment response. However, only a small subsample completed the 3-month follow up, and it was suggested that booster sessions following acute HRT treatment should be provided to support any gains made.

Shareh (2018) randomized patients with DSM-5 trichotillomania to either 8 weekly sessions of a form of therapy involving “metacognitive methods” combined with habit reversal (MCT/HRT) or a wait list control group. The analysis focussed on the 29 completers and found significant differences between the groups on a variety of standardized outcome measures related to hair pulling severity (MGH-HPS; Y-BOCS-TM; self-monitoring) as well as depression (BDI-II), anxiety (BAI); self-esteem (RSES); and global assessment of functioning (GAF), from pre- to post-treatment. Due to the combined therapeutic approaches and the use of a wait list control only, one must be cautious about the conclusions drawn about the effect of HRT.

There were two further studies that were excluded in the process, but the reviewers felt were still important to report upon for completeness. The first, conducted by Flessner et al. (2008) conducted a pilot study in which 6 adult participants with either Trichotillomania or Chronic Skin Picking were allocated to either Acceptance and Commitment Therapy (ACT) followed by HRT, or HRT followed by ACT. Although the term “randomly” was used in the procedure, the “non-concurrent, multiple-baseline design” in the 6 participants involved two treatments that overlapped to such an extent that it was not possible for the reviewers to identify meaningful between group comparisons bearing on the efficacy of HRT.

The second study by Rogers et al. (2014) sought to address questions regarding a two-step model of care for Trichotillomania by only including those classed as “non-responders.” Although participants were “randomly assigned” to an immediate Step 1 condition, comprising “10 weeks of free access to StopPulling.com, consisting of assessment, intervention, and maintenance modules,” or to waitlist, patients in both groups then had the option of accessing HRT, which itself was not randomized.

#### Meta-Analyses

In addition to the individual RCTs, two meta-analyses were identified by the literature search (Bloch et al., 2007; McGuire et al., 2014). Both appeared to demonstrate the benefits of HRT within the meta-analyses, but the authors drew attention to the serious limitations of the small number of studies that existed. For example, the lack of available RCTs that examine interventions targeted at youth with Trichotillomania. No single validated clinical instrument was used consistently across the research to measure severity and improvement of Trichotillomania symptoms. In addition, the meta-analyses indicated a lack of long-term follow up to establish the long-term durability of initial treatment gains, and lack of acknowledgment of comorbidities.

#### Habit Reversal Therapy in Excoriation Disorder

Only two RCTS were identified, alongside several case studies of HRT for skin-picking behavior. As the diagnosis of excoriation disorder was only defined in the last few years, neither RCT made a formal diagnosis of Excoriation Disorder, but instead relied on self-reports of skin picking.

Please refer to Table 2 for a summary of the randomized controlled studies.

**Table 2:** Randomized controlled trials of habit reversal therapy in excoriation/skin picking.

**Study/Country**	**Assessment of disorder**	**Excluded comorbidity**	**Interventions**	**No. HRT/BT sessions**	**N**	**Mean age (Years)**	**Outcome measures**	**Duration of the trial (baseline to end point)**	**Follow-Up, after end point?**	**ITT analysis? Yes/No**	**Outcome at endpoint**	**Treatment responders (%)**
(Teng et al., 2006)/USA	Self-report of picking skin at least 5 times per day over a 4-week period resulting in either social impairment or physical injury.	N/R	1. HRT 2. Wait List	3	25	24 years (SD, 11.6)	Self-monitoring cards; photographs; Social Validity Rating Scale; TEI-SF	Approximately 5 weeks	Yes, 3-month post treatment	No	HRT better than wait list on between group analysis of self-reported skin picking (*p* < 0.01).	N/R
(Moritz et al., 2012)/Germany	Self-report “skin picking.”	None	1. HRT self-help manual 2. Decoupling self-help manual	N/R	70	1.28.37 2. 29.54	M-SPS; BDI-SF	4 weeks	No	Yes	HRT better than decoupling on between group analysis using ITT on the M-SPS (*p* = 0.04) and the completer analysis (*p* = 0.03).	N/R

*BT, Behavioral Therapy; BDI-SF, Beck's Depression Inventory Short Form; HRT, Habit Reversal Therapy; ITT, Intent to Treat Analysis; M-SPS, Modified 10-item Skin Picking Scale; TEI-SF, Modified Treatment Evaluation Inventory-Short Form; TAU, Treatment as Usual; N, Number; N/R, Not recorded*.

#### Individual Randomized Controlled Trials

Teng et al. (2006) randomized college aged students, 1 male and 24 females, to either 3 sessions of HRT delivered over approximately 3 weeks or a waiting-list control group. Due to dropouts during the course of the study, the final sample consisted of 19 females. Analysis of completers indicated significant benefits for the HRT group on self-reported measures of skin picking Photo rankings were also conducted, whereby those in the HRT group were ranked as being significantly more damaged in pre-treatment photos compared to post-treatment and follow-up. Although the control group also reported a significant decrease in skin picking between pre-treatment and follow-up, photo rankings of the skin picking showed no significant differences.

Moritz et al. (2012) randomized 70 predominantly female adult patients to Decoupling, deemed a “variant of HRT,” vs. HRT. Both treatments were administered in the form of self-help treatment manuals. This therefore made it difficult to quantify the amount of treatment received. Those treated with HRT showed significantly greater improvement on the Modified 10-item Skin Picking Scale (*p* = 0.04) compared to those in the Decoupling treatment group on an intent to treat analysis. Similarly, analysis of the completers supported the significant findings (*p* = 0.03). However, it is worth noting that no follow up measures were taken, thus there was a lack of evidence for long term benefits of the intervention.

There were also 6 case studies/series identified and 3 literature reviews.

#### Habit Reversal Therapy in OCD

The majority of published studies reporting on HRT for OCD recruited patients with Tourette Syndrome/Tic disorders as the primary disorder and OCD as a comorbidity; these studies will be discussed in more detail below. A single RCT was identified but excluded on the basis of being a poster abstract only (Rojas and Gair, 2017). The abstract reported the outcome of a 12-session group intervention, which included habit reversal, on symptom scores in a pediatric OCD sample and stated that the group intervention was effective. The substantive peer-reviewed report could not be found. One literature review was identified (Coffey and Rapoport, 2010) which reviewed OCD and Tourette's Disorder, and spoke very briefly regarding habit-reversal being used in the treatment of Tics.

#### Habit Reversal Therapy in Other Disorders With OCD/OCRD as a Secondary/Comorbid Disorder

We found 3 studies of HRT for Tourette Syndrome/Tic Disorder, with OCD listed as a comorbidity. One was an RCT (Seragni et al., 2018), another a pilot study (Woitecki and Döpfner, 2012), and the last a case study (Bryson et al., 2010). The full report of the pilot study was available in German only, so was excluded. However, it is worth noting that the available English translated abstract reported that positive results were found in the effect of HRT on comorbid symptoms, including OCD. Both the RCT and case study did not appear to measure OCD symptoms and so were excluded. It is also worth noting the study by Moritz and Rufer (2011), (Table 1), who used the OCI-R to measure comorbid obsessive-compulsive symptomatology in participants with trichotillomania and found a form of treatment including elements of HRT improved obsessive-compulsive symptoms on an intent to treat analysis.

We additionally screened a subsample of 15 identified literature reviews (Du et al., 2010; Jankovic and Kurlan, 2011; Robertson, 2012; Kurlan, 2014), to further ensure there were no overlooked RCTs on the effect of HRT on comorbid OCD symptoms. Within one of the literature reviews, a single case example was reported (Sulkowski et al., 2013) of a 15-year-old female with OCD and Trichotillomania treated using combined elements of ERP and HRT. Post treatment, her OCD symptoms reduced by 59% and Trichotillomania symptoms by 74%.

#### Habit Reversal Therapy in Hoarding Disorder and Body Dysmorphic Disorder

No published research into HRT and hoarding disorder, nor BDD was found.

### CONSORT Evaluation

Table 3 presents the CONSORT evaluation of all 10 RCTs identified through the literature search; 2 falling under the primary diagnosis of Excoriation (described as “skin picking”), 8 with the primary diagnosis of Trichotillomania. Each paper was assessed on 25 standard items. The two raters reached agreement on all ratings apart from items 20, 21, and 22 for one study (Shareh, 2018). For these three ratings, the lead author's (ML) ratings were used.

**Table 3:** CONSORT evaluation of reporting of 10 RCTs investigating Habit Reversal Therapy in OCRDs.

**Study**				(Shareh, 2018)^*^	**(Rahman et al., 2017)**	**(Keuthen et al., 2012)**	**(Moritz and Rufer, 2011)**	**(Woods et al., 2006)**	**(Dougherty et al., 2006)**	**(Ninan et al., 2000)**	**(Azrin et al., 1980)**	**(Moritz et al., 2012)**	**(Teng et al., 2006)**
**Disorder**				**Trichotillomania**	**Trichotillomania**	**Trichotillomania**	**Trichotillomania**	**Trichotillomania**	**Trichotillomania**	**Trichotillomania**	**Trichotillomania**	**Excoriation**	**Excoriation**
Title and abstract	Title and abstract	1a	Identification	○	●	●	○	○	○	◒	○	○	○
		1b	Structured Summary	◒	◒	●	◒	◒	◒	◒	◒	◒	◒
Introduction	Background and Objectives	2a	Background and explanation of rationale	◒	◒	●	●	◒	◒	◒	◒	◒	●
		2b	Objectives/ Hypotheses	◒	●	●	●	●	◒	◒	◒	●	●
Methods	Trial Design	3a	Trial Design	◒	◒	◒	◒	○	◒	◒	◒	◒	◒
		3b	Changes to method	○	○	○	○	○	○	○	○	○	○
	Participants	4a	Eligibility	●	●	●	●	●	●	◒	○	●	●
		4b	Settings and locations	◒	○	◒	◒	◒	○	◒	●	●	○
	Interventions	5	Interventions	●	◒	●	◒	◒	◒	●	●	●	◒
	Outcomes	6a	Outcome measures	●	◒	◒	◒	◒	◒	◒	◒	◒	◒
		6b	Changes to trial outcomes	○	○	○	○	○	○	○	○	○	○
	Sample Size	7a	Sample Size Determined	○	◒	○	◒	○	○	○	○	○	○
		7b	Interim analyses and stopping guidelines	○	○	○	○	○	○	○	○	○	○
Methods (Randomization)	Sequence Generation	8a	Method for randomization	○	○	○	●	○	○	○	◒	○	○
		8b	Type of randomization	○	○	○	◒	○	○	○	○	○	○
	Allocation concealment mechanism	9	Mechanism for randomization	○	○	○	◒	○	◒	○	○	○	○
	Implementation	10	Who implemented	○	○	○	○	○	○	○	○	○	○
	Blinding	11a	Who blinded	○	◒	○	○	◒	◒	●	○	○	◒
		11b	Similarties in intervention	○	○	○	○	○	○	○	●	●	○
	Statistical Methods	12a	Stats for primary and secondary	●	●	●	◒	◒	●	●	◒	●	○
		12b	Additional analyses	○	◒	○	◒	○	●	●	○	○	○
Results	Participant Flow	13a	No. assigned, received, and analyzed	◒	●	●	●	◒	◒	◒	●	●	◒
		13b	Attrition	◒	○	◒	●	◒	◒	●	●	●	◒
	Recruitment	14a	Dates of recruitment and follow up	◒	◒	◒	◒	◒	◒	○	◒	○	◒
		14b	Why trial ended or stopped	○	○	○	○	○	○	○	◒	○	○
	Baseline Data	15	Baseline demographic and clinical characteristics table	◒	●	●	●	◒	◒	○	●	●	○
	Numbers Analyzed	16	No. in each analysis	○	◒	●	◒	◒	◒	○	◒	●	◒
	Outcomes and Estimation	17a	Results for each group, estimated effect size and precision	◒	◒	◒	●	◒	◒	◒	◒	●	◒
		17b	Binary outcomes absolute and relative ES recommended	○	○	○	○	○	○	○	○	○	○
	Ancillary Analyses	18	Other analyses	○	○	○	◒	◒	◒	◒	○	○	◒
	Harms	19	Harms and unintended effects	○	○	○	○	○	○	◒	○	○	○
Discussion	Limitations	20	Trial limitations	◒	◒	◒	●	◒	◒	◒	◒	●	◒
	Generalizability	21	Generalizability	◒	◒	◒	◒	◒	◒	◒	◒	◒	◒
	Interpretation	22	Interpretation consistency	◒	◒	◒	◒	◒	◒	◒	◒	◒	◒
Other Information	Registration	23	Registration no and name of trial registry	○	○	●	○	○	○	○	○	○	○
	Protocol	24	Where full protocol can be accessed	◒	○	◒	◒	◒	◒	◒	◒	○	○
	Funding	25	Sources of funding and other support	●	◒	●	◒	◒	◒	○	○	○	○

◒, 0; ◒, 1; ●, 2;

* *RCT Section of Study Evaluated Only*.

A comprehensive evaluation of all the limitations of the individual studies was beyond the scope of this paper; instead, an overview of the studies' *main limitations* is described. In general, the studies did report some level of background and explanation of rationale, research objectives, overviews of interventions, participant flow information, and study limitations, generalizability, and interpretation consistency. There was also reasonably thorough and adequate reporting of eligibility criteria of participants across the studies.

However, there were common weaknesses across many if not all the RCTS in identifying the study as an RCT, reporting the methods used for randomization, blinding, sample size determination, statistical analysis, recruitment, process, outcome evaluation, changes to the methodology, interim analysis, stopping guidelines, as well as reporting about who implemented the assessment or treatment, the similarities of comparator interventions, why the trial was ended or stopped, reporting of harms or unintended effects, registration number and name of trial registry, and where the full protocol can be accessed. Of these limitations, failures to identify the study as an RCT, report on power calculations, and report adverse effects/events are arguably critical.

### Areas for Quality Improvement in HRT Trial Design

#### Identification as an RCT

Although all the studies evaluated were identified as an RCT by the author, this was often difficult to establish. Only two studies reported that they were an RCT within the title (Keuthen et al., 2012; Rahman et al., 2017). One further study reported that it was a randomized controlled study within the abstract (Ninan et al., 2000), whilst the remainder of studies only reported this within the methodology.

#### Information on Randomization

Apart from two studies (Azrin et al., 1980; Moritz and Rufer, 2011), the studies were seriously deficient in providing information regarding the randomization process; the method, type, and mechanisms for randomization.

#### Blinding Procedures

Only Ninan et al. (2000) adequately fulfilled this criterion, stating who was blinded to the treatment condition at pre and posttreatment, and how blinding was maintained. Further, they stated the instances where blinded ratings were not conducted. Some RCTs partially fulfilled the criteria (Dougherty et al., 2006; Teng et al., 2006; Woods et al., 2006; Rahman et al., 2017), for example briefly stating that particular raters were blinded, but giving very little further information, if any. Those remaining did not reference blinding to any extent.

In regards to designing comparison interventions that were similar to the experimental treatment, only two studies fully fulfilled this criterion (Azrin et al., 1980; Moritz and Rufer, 2011) by clearing drawing comparisons between the conditions of the experiment. The remainder of studies did not fulfill this criteria at all.

#### Sampling Issues

We consistently found that studies did not justify their sample size. Rahman et al. (2017) presented a power analysis to determine the necessary sample size to observe a moderate or greater effect size. The only other study that partially fulfilled this criterion was Moritz and Rufer (2011) who stated that recruitment was stopped after a specified time period.

#### Changes to Method and Trial Outcomes

None of the studies reported any changes, or lack thereof to the methodology from that defined in their protocol. The CONSORT statement suggests that a change could be due to a “disappointing recruitment rate”; most studies reported some level of dropouts from their original samples that may have led to some change in their method but this was not reported.

The same was found for the item of changes to trial outcomes; no studies identified whether there had been any changes to the outcomes, including data collection or methods of analysis.

#### Why the Trial Was Ended or Stopped

Only Azrin et al. (1980) partially fulfilled this criterion.

#### Harms and Unintended Effects

Only Ninan et al. (2000) reported information relating to harms and unintended effects, and this was limited to the adverse effects of the pharmacotherapeutic condition, and the impact that this had on the results. Some studies, for example, Dougherty et al. (2006) reported within the methodology that should any side effects be experienced, participants were instructed to contact the study's physician investigator, but no numbers reflecting this were reported.

#### Other Information: Registration and Protocol

Only Keuthen et al. (2012) reported the registration number and name of the trial registry, and none of the studies provided information regarding where the full study protocol could be accessed and information regarding sources of funding and other support.

## Discussion

This systematic review was designed to establish the current research base relating to the use of HRT for patients suffering with OCRDs, and to evaluate the quality of the reporting in any RCTs identified using CONSORT criteria. A few systematic reviews have investigated HRT for individual OCRDS. For example, Lochner et al. (2017) reviewed the treatment options for skin-picking disorder. Although acknowledging the benefits of behavioral treatments, the authors drew attention to the sparse evidence base, and a need for consensus on symptom measures. Another review by Selles et al. (2016), drew similar conclusions, again referencing the lack of studies of treatments for excoriation. As far as we are aware, this is the first systematic review conducted specifically on the efficacy of HRT across all OCRDs, rather than the broader class of behavior therapies (e.g., CBT, ERP), which only *sometimes* includes HRT. A total of 10 RCTs were identified as incorporating HRT to treat an OCRD—specifically Trichotillomania and Excoriation. Four studies reported the exclusive use of HRT as the experimental intervention. Of the 10 RCTs, 6 of these had some form of credible control group for comparison (e.g., an alternative treatment, including a pharmacotherapeutic intervention), whereas 3 used a wait-list control group which is not acceptable. One RCT (Dougherty et al., 2006) studied HRT in treatment non-responders, so conclusions about the efficacy of HRT are limited.

In the RCTs identified, there was no clear evidence *against* the use of HRT; rather all the studies showed some evidence of a decrease in symptoms in the experimental groups receiving the HRT (regardless of the form in which this was delivered) relative to the control group. Thus, the findings broadly support the hypothesis that HRT could be an effective treatment intervention and warrant further investigation. However, no firm conclusions can as yet be drawn owing to the methodological limitations identified in this review.

Habit Reversal Therapy was originally developed as the Habit Reversal Procedure for treating nervous habits and tics. Thus far, the evidence hints that HRT may be an effective treatment for Excoriation and Trichotillomania and provides scope to expand research to explore the clinical efficacy of HRT across the full range of OCRDs, including OCD, BDD and hoarding disorder, in the form of well-designed RCTs. This approach would complement the emerging neurosciences research demonstrating biologically-based biases away from goal-directed control and toward habitual responding in the pathophysiology of OCD.

According to this research, anatomical overlaps have been demonstrated between the neural substrates of habit formation and the pathophysiology of OCD, converging in the cortico-thalamo-striatal neurocircuitry (Graybiel and Rauch, 2000; Burguiere et al., 2015). These observations led to a recent series of experiments investigating the role of habit learning in OCD, which demonstrated that patients with OCD show evidence of a shift *away from* goal-directed control and *toward* habitual responding, in paradigms variously testing slips of action during a rewarding game (Gillan et al., 2011), decision-making tendencies (Voon et al., 2015) and avoidance of electric shocks (Gillan et al., 2014). Further research by Gillan et al. (2015) using functional brain imaging of OCD patients whilst performing a habit learning task, reported dysfunctional hyper-activity in the caudate nucleus and medial orbitofrontal cortex. The caudate nucleus plays a key role in goal directed learning. Therefore, the finding that caudate hyperactivity was associated with self-reported urges to perform avoidance habits suggested that the habitual responding seen in the patients with OCD resulted from a primary failure in goal-directed control over actions. Together, these studies provide growing evidence of a selective deficit in goal-directed control over actions, resulting in biases toward performing habits, that may contribute to the symptomatology of OCD.

Habits are unlikely to completely explain the performance of compulsions, even in chronic states. However, by helping the individual to break the link between the stimulus (exposure to the cue to perform the compulsion) and response (the compulsion itself), HRT may theoretically be used alongside traditional CBT techniques such as exposure and response prevention as a method to extinguish compulsions more readily. Hypothetically, by weakening the habit, the individual may be able to exert greater instrumental control over the compulsive behavior, rendering it more amenable to conventional CBT techniques. This hypothesis could be tested using randomized controlled trial methodology comparing CBT with or without adjunctive HRT delivered as a prior intervention.

Throughout the review, we found evidence of benefit for “variants” of HRT, for example “movement decoupling” (Moritz and Rufer, 2011). The authors argue that HRT and decoupling may each “stop the dysfunctional movements by actively interfering at the motor level.” Our work raises questions about the clinical importance of modifications that can be made to HRT, such as the added value of using a combination of HRT and another behavioral intervention.

The results of the CONSORT evaluation indicated that no studies thus far have filled a “good enough” standard against these criteria and highlight the need for improved trial design and reporting in the key areas of randomization, blinding, and sampling procedures. Some of these deficits have been highlighted in other evaluations of RCTs of CBT (King et al., 2017). However, it is important to note that research in this field can still be considered in its infancy, and the existing studies, have provided a vital starting point.

Key areas for improvement to aid future research were identified. We identified a concerning lack of research using children and/or adolescents, which represents a vital age range to target given the early onset for many of these disorders (Diefenbach et al., 2000; Walsh and McDougle, 2001). There is also a clear need for agreement on the primary outcome measures for many of the disorders assessed. For example, neither of the RCTs of skin picking used the same scale, and there was a reliance on self-monitoring in some form for both Excoriation and Trichotillomania. There was most consistency in the measurement of Trichotillomania, with the Massachusetts General Hospital Hairpulling Scale being used throughout the majority of the studies as one of many outcome measures. In addition, greater attention needs to be paid to those comorbid disorders that commonly occur with OCRDs and that may impact on treatment outcomes (Sulkowski et al., 2013). Evaluation of the impact of treatment on the medical complications and psychosocial consequences of these disorders is also important- for example in Trichotillomania (Frey et al., 2005) as well as social alienation and depression.

## Limitations of the Review

We were unable to include articles unavailable in the English language. Although this did not appear to impact our review bar one paper (as acknowledged, Woitecki and Döpfner, 2012), it is possible that there is further work that was not identified using our method. In addition, by limiting the choice of databases searched, we may have missed some additional published research. However, review of the Cochrane database provided at least partial confirmation of the main search findings, and did not result in the identification of any further articles meeting the inclusion criteria. There is a further possibility that relevant research was not identified due to publication bias. For example, we became aware of a case report by Dillenburger (2006) that was not identified by our search and that used Habit Reversal Therapy as a successful treatment for OCD. It is possible that, as we screened the title and abstract only for key words and phrases, some research incorporating HRT was missed as reference to it was only made in the methodology section. In regards to the CONSORT evaluation, there were some discrepancies between the reviewers on the quality ratings, however, the approach taken to resolve this endeavored to ensure that all ratings were as consensual as possible.

## Conclusion

So far, the evidence supporting the effectiveness of HRT in OCRDs is limited to a few studies of Trichotillomania and Excoriation. The existing studies are limited in terms of quality of study design and reporting. Our review highlights the need for well-designed studies to be conducted in the appropriate age groups across all the OCRDs. Such studies should measure key primary and secondary outcomes using well matched controls and blinded raters.

## Author Contributions

All three authors contributed to the paper searching, writing, analysis, and conclusions. ML and DM performed the CONSORT evaluation.

### Conflict of Interest Statement

NF reports personal fees from OTSUKA, LUNDBECK, ABBOTT, SUN Pharma, TAYLOR AND FRANCIS, ELSEVIER; personal fees and non-financial support from RANZCP, WILEY; grants from NIHR, WELLCOME; grants and non-financial support from EU, ECNP, SHIRE; non-financial support from BAP, WHO, CINP, ISAD, RCPSYCH, INTERNATIONAL COLLEGE OF OC SPECTRUM DISORDERS, IFMAD, MHRA; other from OXFORD UNIVERSITY PRESS, all outside the submitted work. The remaining authors declare that the research was conducted in the absence of any commercial or financial relationships that could be construed as a potential conflict of interest. The handling Editor declared a past co-authorship with one of the authors, NF.
