# Supplementary material for: Habit Reversal Therapy in Obsessive Compulsive Related Disorders: A Systematic Review of the Evidence and CONSORT Evaluation of Randomized Controlled Trials
Source: Front Behav Neurosci. 2019 Apr 24;13:79. doi: 10.3389/fnbeh.2019.00079 (PMC6491945; doi:10.3389/fnbeh.2019.00079)
Supplement: Supplementary file 1 [file Data_Sheet_1.docx]

**Appendix 1**

Search Terms Used for Literature Searches:

“habit reversal”

“habit-reversal”

“habit reversal therap*”

“habit reversal training”

“habit-reversal training”

“habit reversal treatment*”

“habit-reversal treatment*”

“OCD”

“obsessive compulsive disorder*”

“obsessive-compulsive disorder*”

“obsessive compulsive*”

“obsessive-compulsive*”

“trichotillomania”

“trichotillomania disorder*”

“hair pulling”

“hair-pulling”

“hair pulling disorder*”

“hair-pulling disorder*”

“compulsive hair pulling”

“compulsive hair pulling disorder*”

“body dysmorphic disorder*”

“body-dysmorphic disorder”

“BDD”

“body dysmorphia”

“hoarding”

“hoarding disorder*”

“hoarding-disorder*”

“extreme hoarding”

“extreme hoarding disorder*”

“hoarding behavio*r*”

“excoriation”

“excoriation disorder*”

“skin picking”

“skin-picking”

“skin picking disorder*”

“skin-picking disorder*”

“compulsive skin picking”

“compulsive skin picking disorder*”
